# Sustainability as an Ethical Principle: Ensuring Its Systematic Place in Professional Nursing Practice

**DOI:** 10.3390/healthcare4010002

**Published:** 2015-12-30

**Authors:** Annette Riedel

**Affiliations:** Faculty of Social Work, Health Care and Nursing Sciences, University of Applied Sciences Esslingen, Flandernstraße 101, 73732 Esslingen, Germany; annette.riedel@hs-esslingen.de

**Keywords:** sustainability, ethical policy, ethical reflection

## Abstract

Alongside the central focus on the persons requiring nursing care in professional nursing practice, the perspective of the sustainability of interventions and the use of materials (for example, nursing aids and hygiene articles) is gaining prominence in nursing decision-making processes. This contribution makes the principle of sustainability concrete and delineates its importance in the context of professional nursing practice and decision-making. It further suggests the development of an ethical policy in order to systematically ensure that sustainability has a place in ethical reflection and decision-making, and describes the elements involved. Finally, a synthesis is made between the importance of the principle of sustainability, suggested ethical policies (system of ethical reflection) as they affect nursing practice and professional reflection, decision-making, and practice.

## 1. Introduction

Professional nursing practice is characterized by an increasingly high degree of complexity. Complexity in the nursing situation arises from specific professional challenges in which decision-making is based on theory-derived practice and a hermeneutic understanding of cases [1]. It is further increased by social and political expectations of the health care system. Thus nursing is expected to respond to currrent social developments (such as increasing numbers of extremely old people or cultural diversity), to patients’ expectations and to economic limitations. Alongside the central focus on people requiring nursing care in professional nursing practice, the perspective of the sustainability of interventions and the responsible use of materials (for example, nursing aids and hygiene articles) is gaining prominence in professional and ethical nursing decision-making processes. Sustainability as a (complementary) ethical/moral principle in making decisions in professional nursing practice is in part a response to ever decreasing resources in health care. Sustainability as a principle in decision making in the context of ethical considerations sensitizes us to the additional responsibilities carried by nurses in their professional practice. Due to their inherent potential for ethical conflict, these complex demands repeatedly lead to moral distress in day-to-day nursing situations. However, because of their high time and personnel requirements, repeated ethical case conferences are often not possible for ethical questions. For these reasons, this contribution considers how the development of ethical policies can be used in repeatedly recurring ethically problematic day-to-day professional nursing care situations requiring some kind of solution. Here, the following questions need to be addressed: can ethical policies ensure ethical professional practice? Which practice-related demands must ethical policies consider in order to initiate ethical reflection and ethically based decision-making? Can ethical policies ensure ethically grounded decision making in the context of ongoing ethical questions related to the demand for sustainability in professional nursing care and provision? Against the background of recurring ethical dilemmas—especially those arising from current social and economic developments—the aim of this contribution is to present ethical policies as a systematized method for making ethical decisions and to analyze and discuss them as a potential, future-oriented approach within the framework of professional nursing practice. Here, the normative orientation focuses on the example of the principle of sustainability as an increasingly important principle in responsible nursing practice, as will become clear in the following.

This contribution is based on three hypotheses:

1. Sustainability as an ethical principle requires more attention in professional nursing practice and decision making due to its growing importance, e.g., in dealing with increasingly scarce resources.
2. In order to sensitize nursing staff to sustainability as an ethical/moral principle in the context of their professional practice, supporting procedures are necessary.
3. Ethical policies which have been developed for specific situations provide professional nurses with a systematized way for applying sustainability as an ethical/moral principle responsibly in those specific situations and to arrive at decisions derived from ethical considerations which are relevant to those situations.

The first step in this contribution will be to elucidate the principle of sustainability and its significance in the context of professional nursing practice and decision making (Section 2). The second part will be concerned with describing the process of creating and justifying the value system of an ethical policy as it relates to ethically based decision making in nursing (Section 3). A synthesis of the significance of the principle of sustainability and a system of ethical reflection—related to professional nursing ethical reflection, decision making, and practice—will be discussed in Section 4.

## 2. Sustainability—A Principle for Responsible Nursing Practice

The concept of sustainability is complex and the term is used in many different contexts, including the context of health care systems, as in Sustainable Healthcare Systems [2]. Sustainability has become a key concept in many discussions and political declarations. Apart from ecological issues, there is also the question of the distribution of increasingly scarce resources. Today, the scarcity of resources is already evident in concrete nursing situations. Further, in the context of nursing and health care, there is the additional factor of sustainably ensuring the financial basis of the system and personnel [3] as well as environmental aspects [4]. It is clear that the principle of sustainability has become significant for professional practice, that is, both in terms of decision making and in guiding practice. Section 2 will be concerned with hypothesis 1.

### 2.1. Sustainability in Professional Nursing Practice

As up to now there have been no clear and concrete basic definitions of sustainability in the context of professional nursing practice and decision making, in the following, a preliminary attempt to provide one will be made. The aim will be to delineate sustainability as a guide to practice in the context of professional nursing practice and ethical decision making. This means placing the principle of sustainability firmly within professional nursing. The intention here is not to provide a fixed and definitive statement, but rather to concretize and approach the subject.

In the German-speaking world, sustainability is most often mentioned in the context of managing resources within the discourse on the environment [5,6]. This means that sustainability is not a static phenomenon. The German word for sustainable (nachhaltig) was first used in the context of forestry and the need to maintain forests for coming generations [5,6,7]. The combination of terms “sustainable development” became popular following the so-called Brundtland Report [5,6]. The World Commission for the Environment and Development [8] coined the oft-quoted and until today the most broadly used and recognized definition: “Sustainable development is development that satisfies the requirements of the present without endangering the ability of future generations to satisfy their requirements” [8]. Alongside the premises of stability and conservation, this linking of two central terms—“sustainable” and “development”—brings a certain dynamism into the context of sustainability. The German discourse on sustainability moreover rests on the so-called “three-pillar model” in which a social dimension is included, and instead of a purely environmental guiding principle also postulates a social one [6,7]. This social perspective is also represented in the NurSus project’s [4] exploratory definition of sustainability: 

“Designing and delivering health care that meets today’s health and health care needs of individuals and populations without compromising the ability of future generations to meet their own health and health care needs; this requires the provision of health care that recognizes and respects the dependence of our health on the earth’s ecosystems, without resulting in unfair or disproportional impacts within society [4]”.

The essence of sustainability also includes the idea that scarce resources can and should be better distributed—a theme that is becoming increasingly relevant in health care. Within the context of nursing, health care systems and health care politics [3], sustainability is gaining relevance on several levels: there are scarce personnel resources and therefore a lack of time; scarce income from contributions and thus a lack of services; and a scarcity of beds bringing with it shorter hospital stays, *etc.* Discussions about allocation, rationing and rationalization are the order of the day.

### 2.2. Sustainability as an Issue in Ethical Considerations and Ethically Grounded Decision Making

It is not entirely clear what significance the principle of sustainability has in the context of ethical considerations in response to conflicts concerning the distribution of resources. Here, the concept of sustainability is not only concerned with scarcity as such, instead it is also concerned with its causes. Sustainability therefore demands accountability and responsibility in practice, looking ahead in anticipation of potential consequences and thus a broadening of perspective—away from the sole perspective of current, present-day practice to the additional perspective of expectable consequences in the future [5].

Thus sustainability as a basic value does not mean, for example, thinking in terms of today’s patients, but rather for all future patients; not in terms of a specific patient on the ward here and now, but for all future patients in the hospital; not only of nursing and nurses today, but rather for the nursing and nurses of tomorrow. Sustainability means addressing current needs and requirements without putting future options for healthcare delivery at risk. Here, the concept of fairness is decisive: we are talking about a fair distribution of health-related goods and services, or to put it more broadly, distribution under conditions of increasingly scarce resources.

An indispensable requirement of sustainability is to think long-term. The challenge is to sensitize people to the dimension of sustainable decisions and to draw attention to the long-term consequences of practices and decisions carried out today. For this sensitization to be effective, it is necessary to analyze, consider and weigh up the potential consequences for health care delivery in nursing education and day-to-day practice and to demand that decisions are oriented towards sustainability. Some examples of this are: what does it mean for the next shift, if there is not enough sterile disposable material available? What does it mean for a hospital if costs are capped? What does it mean for the quality of life of nurses and patients, for human dignity, if the day-to-day distribution of nursing resources is marked by conflict? What becomes clear is that in essence, sustainability is a practical principle as well as an ethical/moral one [6]! With this in mind, the question arises as to what significance these sustainability discourses, discussions, and definitions have in the context of ethics and ethical considerations in nursing practice. In the following, ethics will be understood as providing a basis for reflection—as systematic as possible—for moral behavior. Values are an essential part of ethical reflection and moral behavior. As a normative discipline, ethics provides a framework for what nurses should do or what their professional responsibilities are.

### 2.3. Sustainability as an Ethical/Moral Principle

Beginning from the premise that in essence sustainability is a guide to practice as well as an ethical/moral principle, the question arises as to how this principle can be concretized and contextualized in respect to ethical reflection and decision making. Here, the question especially arises as to which normative dimensions stand for the principle of sustainability and the striven-for sustainable developments in professional nursing practice. These normative dimensions in turn can be seen as delineating the complex principle of sustainability but also as providing a framework for ethical reflection and decision making in nursing. In this, I am not proposing a “catalog of duties” [7] or ethical postulates, rather I am attempting to delineate sustainability as an ethical principle/guiding principle in nursing practice. This means clarifying which ethical founding principles, which normative facets, which practice-relevant and decision-making values and moral duties are inherent in sustainability, and which, in their turn, could form a basis for decision-making—e.g., in decisions concerning the allocation or rationing of resources or in any day-to-day decisions involving an extensive use of resources. From the foregoing discussion, it follows that sustainability and sustainable development can be associated with the following moral norms and values: justice (distributive justice or fairness, questions about the distribution of health-relevant goods and services); responsibility (professional, moral and causal responsibility); and quality of life (subjective and objective assessments of quality of life). These values can in turn be interpreted, described and weighted in the most diverse ways, according to the (normative) situation. At this point, the normative framework of the principle of sustainability is primarily intended, with reference to the foregoing discourse on sustainability and its significance for sustainability in professional practice. Accordingly, the principle of sustainability as it relates to professional nursing practice can be delineated as follows: sustainability as an ethical/moral principle requires that decisions should be made that not only focus on current actions but also anticipate potential, expectable consequences. Sustainability as an ethical/moral principle in professional nursing is committed to the relevant normative dimensions of justice, responsibility, and quality of life.

The following section describes the aims and steps in developing an ethical policy which provides a procedure for ensuring that decision making is systematized, ethically considered, and ethically grounded. A synthesis related to professional nursing reflection, decision making and practice will be discussed in Section 4.

## 3. Ethical Policies—A Procedure for Systematized, Ethically Grounded Decision Making

Increasingly complex nursing practice depends on systematized procedures and methods that help nurses in recurring day-to-day situations which require ethical decision making, assist them in structuring their decision-making processes and ensure that decisions are made within the framework of ethical counselling. In the context of ethical counselling, ethical case conferences and ethical policies are especially relevant and authoritative [9,10,11,12]. In the following, the main emphasis will lie with ethical policies, as these can be developed and implemented in every type of organization and every setting. Section 3 will be concerned with hypotheses 2 and 3.

### 3.1. Relevance and Clarifying Definitions

In the context of ethical counselling, ethical policies provide a recognized, supportive, and systematized procedure for making ethical decisions in individual cases. They have been developed as an aid to making ethical decisions in situations that recur repeatedly [1,11,12,13,14,15]. Their main aim is to provide a guide to ethical reflection and to help make decisions which are transparent and ethically founded (also for non-participants). When considering ethical reflections which are situationally relevant and supported by ethical policies, we are talking about a conscious, ideally structured analysis of the nursing situation, looking at its morally relevant content and at the guiding values and value orientations which affect the decision itself. For procedures involving ethical decision making, this means that ethical policies not only open the way for high-quality ethical analysis and decision making (methodological approach) but should also provide clear, normative justifications in the form of normative criteria (substantive normative criteria for ethical analysis) [16,17].

There follows an examination of concrete definitions of what is understood by ethical policies in the health care system. The emphasis will lie on the aims which are associated with the development of ethical policies, on the factors which support their development, and on their ethical/normative guidance in connection with situationally relevant ethical reflection and ethically grounded decision making. The second step will be to form a link to the principle of sustainability by creating a synthesis between the presented basic principles and the required normative framework. In the following, sustainability—as an ethical/moral principle in professional nursing practice—will be treated as a entral normative criterion in the selected system of ethical consideration, reflection, and decision making: the ethical policy.

Concrete definitions and descriptions of ethical policies can be found for example, in two recognized associations in the area of medical ethics in the German-speaking world: the German Academy for Ethics in Medicine (AEM) and the Swiss Academy for Medical Sciences (SAMW). In the following, these two definitions will be set out. The two definitions are explicitly concerned with ethical policies and are not extended to include “guidelines for decisions”, which could similarly have ethical implications [18]. Thus, the German Academy for Ethics in Medicine (AEM), in its *Standards for Ethical Counselling in the Health Care System,* defines ethical guidelines/policies as “recommendations for practice which are derived from recurring situations (...) and serve as an orientation in decision making in individual cases [9].” For medical practice, this means that the situation involved, because of its complexity, provokes repeated ethical dissent in the day-to-day provision of health care so that ethical guidelines/policies can aid decision-makers and those concerned to follow a systematized path for ethical reflection in that situation. In the *Recommendations for Creating Ethical Policies in Health Care Organizations* from the same association (AEM), this type of systematized orientation in the context of repeatedly arising ethical conflicts and questions is seen as helpful inasmuch as adherence by all those involved in the decision-making process can improve the quality of the decisions made and can lead to a reduction in ethical distress [12]. To meet these requirements, ethical policies must provide a defined corridor for decision-making and action which corresponds to currently valid legal norms and represents the current (medical and nursing) ethical state of research. In this way, by facilitating ethically systematized, balanced, and grounded decisions, ethical policies ensure professionality in ethical decision making and resulting actions as well as providing justification on the grounds of moral legitimacy and legal validity [12]. Hence, ethical policies which are oriented towards repeatedly recurring ethical questions—independent of individual cases—provide concrete support and structure for ethically relevant consideration and decision-making in the specific situation. Through their structured content and normative framework, ethical policies are intended to contribute to confident decision making because the decisions so made are based on the delineated aspects, criteria and values; in this way, they are intended to guarantee the best possible justification. In this process, ethical policies do not forestall decision making, but rather provide a system and structure for decision making by setting out relevant ethical bases for consideration and reflection.

The Swiss Academy for Medical Sciences (SAMW) describes ethical policies thus: “In ethical policies, repeatedly recurring problems or conflicts of values are dealt with (...) ethical policies include content-related aspects, worked-through ethical justifications as well as an explicit consideration of values. In so doing, they take into account the specific challenges of the institution [19].” In this definition it is also clear that developing facility-specific and situationally relevant ethical policies makes sense when conflicts of values arise repeatedly. This means that this instrument offers multi-disciplinary teams or groups of affected and involved persons systematized support for deciding which is the ethically best and most appropriate decision: “(…) it combines the relevant ethical arguments in a structured way, thereby guiding the persons involved through the decision process [19].”

### 3.2. Aims of Ethical Policies

The central aim of ethical policies is thus to clarify the values involved in the ethically critical situation which has led to conflict in the team (note that this is not the same as a dispute about technically correct practice). This means identifying the values which have caused moral distress in one or more members of the team. The overarching aim of ethical policies is to support ethically well-grounded decisions. Responsible and scientifically founded ethical policies (which also take into account findings from related disciplines) make an important contribution to professionalization and quality assurance in decision making and subsequently lead to improved quality in health care. The systematization of ethical reflection and ethically grounded decision making has become obligatory, given the increasingly complex decisions, decision constellations, and situations in nursing [12]. A policy-based procedure is intended to increase the efficiency of the decision-making process [12] and ensure a unified approach to recurring ethically conflicted situations and ethical decision-making processes. Through concretization and systematization, ethical policies contribute to equal treatment for equal cases [20,21].

### 3.3. Development, Systematization and Areas of Application

As regards the development of ethical policies, their function in providing a basis for discussion and aid to orientation should be emphasized. This allows their genuine purpose—to systematize ethical reflection and open the way for and support ethically grounded opinion—to be constructively and effectively fulfilled. In order to fulfil the above-mentioned aims, the development of ethical policies places specific demands on the conditions and quality characteristics of the process itself as well as its contents [12,16]. Optimally, ethical policies would be created by and with the persons involved, in the settings in which the ethically critically situations repeatedly arise and cause ethical distress and ethical dilemmas. A situationally relevant development process demands the integration of several ethical and professional perspectives to do with questions about immanent values and anticipated balancing of values. Here, a discursive process which balances values and well-grounded opinions is necessary [13,19,22,23]. The central elements are: phases in situation-based analysis and consideration of technical and professional aspects; phases in interdisciplinary reflection, consideration of values and concretization, the formation of opinions and finally building consensus. A consistently discursive and cooperative procedure ensures that multiple perspectives and values are taken into account, supports reflection and consensus-building processes, and thus gives weight to the final decision, facilitating its acceptance and implementation.

The Recommendations for Creating Ethical Guidelines/Poicies in Health Care Organizations [12] draws attention to the following points which have to be clarified:

- How has the organization dealt with the recurring ethical question in the past?
- What expectations are associated with the creation of an ethical policy?
- Who is the concrete target group for the ethical policy?
- Which moral values can be identified?
- What legal requirements have to be taken into account?
- Which professional, technical, scientific, and evidence-based premises are important?

It is important to specify the relevant contents, areas of application, the situational context, and the target groups for ethical policies. Ethical policies demand “a set of normative criteria based on an explicit ethical justification”. This is “a requirement of any ethical analysis [16].” Here, the significance of a normative foundation and the orienting function of ethical policies becomes evident; these must be clarified in the development process.

In order to be able to realize structured and systematized decision making, so as to be able to offer a practice-relevant orientation for individual decisions, ethical policies include supporting elements which operationalize the relevant steps in the decision-making process. This “structured methodical approach for applying the resulting normative criteria [16]”—in the example of sustainability—ensures that the team applying it has a situationally relevant, stepwise, transparent and consistent procedure. In order to ensure that the ethical-policy-based, decision-making process is transparent and logically grounded, it is usual to develop decision-making algorithms [11,13,15]. These provide for a structured and systematic decision-making process without prescribing any decision in advance. They delineate the ethical/moral framework; the decision is then made according to the situation and taking into consideration the ethical/moral orientation provided and any consequences that could arise from specific practices.

This means that in general, ethical policies include the following aspects and elements [12]:

- Questions and definitions concerning the area of application
- Statement of the values and principles involved
- Criteria for decision making

The binding nature of the use of ethical policies (not the decision!) rests on the ethically critical situation for which the ethical policy was developed—in and with the team. Within this article, the area of application is the requirement for and striving towards responsible practice by professional nurses in the area of sustainability. The concrete necessity for ethical policies is derived from the need to make sure that decisions are made which are ethically grounded—all within the complex field of taking into account sustainability in professional practice and at the same time respecting patients’ individual wishes, needs, and requirements. The concrete form of ethical policies must anticipate any ethical values, principles, and conflicts of value which are potentially involved. Thus ethical policies must be able to stand up to the following questions: “Does the policy involve a potentially controversial moral or social issue?” “Could the policy trigger moral distress and/or conflict amongst staff/patients/families [24]?”

Alongside the requirements for validity and binding character, ethical policies also need information as to their period of validity and when a revision may become necessary (e.g., potential changes in the law, relevant research findings, changes in expert standards and guidelines). It is also essential to clearly delineate the limitations of the ethical policy in order to avoid false expectations. Here, especial focus is on the recommendatory character of decisions so made, as well as their situational relevance. Moreover, in their application they are not to be seen as blueprints [13], nor are they a set of “cookbook ethics” [18]; rather they are a tool to facilitate weighing up of situational factors and ethical reflection. This means: the decision-maker’s responsibility to reflect on the ethical situation remains [11,15]! 

The degree of practice-orientation, applicability, practicability and acceptance (as a practice-related quality standard) of ethical policies can only be assessed in practice and should be categorically and conscientiously considered and evaluated [13,16,23,25].

In the following, an area of application of a possible ethical policy related to the principle of sustainability in professional practice will be sketched, with special reference to the normative framework.

## 4. Sustainability—A Principle of Ethical Reflection and Decision-Making Processes in Professional Nursing Practice

In the following, sustainability as a principle of responsible nursing practice will be described as a central, normative criterion in an ethical policy—a selected systematized method for ethical consideration, reflection, and decision making. In accordance with the requirements for the contents and design of ethical policies described above, central keystones and implications for the development of ethical policies will be presented. Because of the fact that ethical policies are not only intended to open the way for systematic procedure (methodological approach), but should also provide a normative basis in the form of normative criteria (substantive normative criteria for ethical analysis) [16,17], the normative keystones are to be generated as central elements in ethical policies. Section 4 will be concerned with hypotheses 2 and 3.

### 4.1. Normative Keystones

By normative keystones we mean moral values and principles which arise out professional ethical standards, for example, the *ICN Code of Ethics* [26], or the *Code of Ethics for Nurses* [27]. So, for example, the ANA, in provision 6, says: “The nurse, through individual and collective effort, establishes, maintains, and improves the ethical environment of the work setting and conditions of employment that are conducive to safe, quality health care [27].” “Responsibility for the health care environment” is here made explicit. In the glossary, a further important principle can be found: that of “environmental justice [27].” This refers to ethical orientation points that are to be seen as central in the context of sustainability. In the *ICN Code of Ethics,* Section 3 (*Nurses and the Profession*) explicitly formulates: “The nurse practices to sustain and protect the natural environment and is aware of its consequences on health [26].“ 

Even though the term “sustainability” is not explicitly used here, it can be seen that nurses have a responsibility for sustainable practice and decision making and that this includes the relevant element of justice. A central aspect, to be found in both ethical standards, is the significance of environmental factors. Against the background of the cited professional ethical standards, and also on the basis of the above discussions on sustainability as a principle of professional practice, the elements of responsibility (for sustainability in professional practice), of (distributive) justice and quality of life (subjective and objective quality of life assessment) can be identified as ethical orientation points and target dimensions in ethical policies for professional nursing practice. Now the concrete description of the important aspect of responsibility generated in Section 2—in respect to environmental factors—will be expanded and set out.

As an ethical/moral principle, sustainability requires nursing decisions that not only focus on current actions but which also focus on potential expectable consequences and which contextualize environmental factors in a responsible manner. Sustainability as an ethical/moral principle in professional nursing is committed to the normative dimensions of justice, responsibility and quality of life.

### 4.2. An Example from Practice

In every specific individual nursing situation, the values and principles of professional nurses exist vis à vis the values of the person requiring nursing care. These values especially include autonomy (based on the right to self-determination). From the starting point that the ethical/moral principle of sustainability is essential for professional nursing practice, it can happen that in particular decision-making situations the values and principles mentioned above bring with them potential conflict: these may be conflicts of interest or conflicts of values. Both the conflict itself as well as the potentially inherent ethical dilemma can in their turn lead to situational moral distress. Now we have an example of a situation in which a professional nurse has to choose responsibly between the just distribution of expensive goods and the self-determination of the patient (his/her autonomous use of these goods) in a clinical setting. It can be seen, for example, that at weekends, a nurse uses expensive disposable flannels in a wasteful way: the patient’s incontinence requires intimate hygiene every two hours following a change of incontinence pads. This leads to an ethical conflict in the form of deciding between exercising restraint in the use of expensive disposable products (responsibility for sustainability), using an alternative cloth flannel, and the autonomy of the patient who may prefer the use of the disposable flannel over the cloth one.

A consequence of the comprehensive use of limited available disposable flannels for one patient may mean that on a particular weekend there are not enough disposable flannels available for other patients (aspect of just distribution); this in turn influences the (subjective and objective) quality of life of patients who may depend on disposable flannels, e.g., because of an infection. In the case of recurring ethical conflicts in the context of sustainability, a relevant ethical policy such as Ensuring Sustainability in Professional Nursing Practice can offer the nursing team support in making ethically founded decisions which conform to the ethical/moral principle of sustainability. In such situations, the development and implementation of the ethical policy can sustainably contribute to reducing moral distress as well as sustainably assuring nursing and service quality.

## 5. Conclusions

Two elements have been central to this contribution: showing the significance of ethical policies as procedures for offering concrete, structured ethical reflection and decision making; and delineating sustainability as an ethical/moral principle in the context of professional nursing practice and making ethically founded decisions [12,15]. Further, this contribution was based on three hypotheses:

1. Sustainability as an ethical principle requires more attention in professional nursing practice and decision making due to its growing importance, e.g., in dealing with increasingly scarce resources.
2. In order to sensitize nursing staff to sustainability as an ethical/moral principle in the context of their professional practice, supporting procedures are necessary.
3. Ethical policies which have been developed for specific situations provide professional nurses with a systematized way for applying sustainability as an ethical/moral principle responsibly in those specific situations and to arrive at decisions derived from ethical considerations which are relevant to those situations.

Section 2 was concerned with hypothesis 1—that sustainability can be delineated as an ethical/moral principle: “sustainability as an ethical/moral principle requires that decisions should be made that not only focus on current actions but also anticipate potential, expectable consequences. Sustainability as an ethical/moral principle in professional nursing is committed to the relevant normative dimensions of justice, responsibility and quality of life” (see Section 2.3). It is clear that sustainability in professional nursing practice—also in the framework of qualifications [28]—has a significant place. Because sustainability is conceived as a values-based ethical/moral principle, its integration into practice and nursing situations requires differentiated ethical reflection and ethically-grounded decision making (see Section 2.1 and Section 2.2). To ensure that this can occur—and at the same time referring to hypotheses 2 and 3—the procedure of an ethical policy was presented. We can assume that this recognized procedure in professional nursing practice [9,11,12,13,14,15,19,25,29] is also practicable as an aid to ethical reflection and decision making in the context of increasing demands for sustainability, and can provide support in nursing practice. In reference to this hypothesis, ethical policies and their aims as a supporting procedure were presented in some detail. The synthesis in Section 4 demonstrated an example of their use in practice and rests on all three hypotheses presented at the beginning of this paper.

The synthesis is provided by the formulation of moral key points for possible ethical policies which, in the light of the increasing importance of sustainability in professional nursing practice, can support ethically well-founded decisions. This means that this contribution, with its inherent deployment of normative key points, using sustainability as an ethical/moral principle in professional nursing practice and in ethical/normative contexts, can be used as the basis for developing a facility-relevant ethical policy; this can be used in specific, recurring situations by the team, to orient and guide nursing practice. The present discussion makes clear: sustainability as an ethical/moral principle is a genuine element in nursing ethical reflection and professional nursing practice and decision making.

## References

[1] Riedel A. (2013). Ethische Reflexion und Entscheidungsfindung im professionellen Pflegehandeln realisieren. Ethik Med..

[2] Fischer M. (2015). Fit for the future? A new approach in the dabate aubout what makes healthcare systems really sustainable?. Sustainability.

[3] Bundesministerium für Gesundheit Ressortbericht. Nachhaltige Entwicklung in Gesundheit und Pflege. https://www.bundesregierung.de/Content/DE/_Anlagen/Nachhaltigkeit-wiederhergestellt/5-Berichte-Reden/2013–02–22-bmg-bericht-nachhaltigkeit-in-gesundheit-und-pflege.pdf?__blob=publicationFile&v=2.

[4] NurSus. http://nursus.eu/wp-content/uploads/2015/03/NurSus-Newsletter-2.pdf.

[5] Bauchmüller M. (2014). Schönen Gruß aus der Zukunft. APuZ.

[6] Pufé I. (2014). Was ist Nachhaltigkeit? Dimensionen und Chancen. APuZ.

[7] Tremmel J., Sturma D., Heinrichs B., Verlag J.B. (2015). Nachhaltigkeit. Handbuch Bioethik.

[8] Report of the World Commission on Environment and Development: Our Common Future. http://www.un-documents.net/wced-ocf.htm.

[9] Vorstand der Akademie für Ethik in der Medizin e.V. (2010). Standards für Ethikberatung in Einrichtungen des Gesundheitswesens. Ethik Med..

[10] Riedel A., Lehmeyer S., Elsbernd A. (2013). Einführung von Ethischen Fallbesprechungen: Ein Konzept für die Pflegepraxis. Ethisch Begründetes Handeln Praktizieren.

[11] Riedel A. (2014). Ethik-Policy Palliative Sedierung.

[12] Neitzke G., Riedel A., Brombacher L., Heinemann W., Herrmann B. (2015). Empfehlungen zur Erstellung von Ethik-Leitlinien in Einrichtungen des Gesundheitswesens. Ethik Med..

[13] Winkler E.C., Borasio G.D., Jacobs P., Weber J., Jox R.J. (2012). Münchner Leitlinie zu Entscheidungen am Lebensende. Ethik Med..

[14] Dreuw H., Bockenheimer-Lucius G., Simon A. Ethikleitlinie zur Behandlung von Zeugen Jehovas und deren Kinder. http://www.ethikkomitee.de/downloads/ethikleitlinie-zur-behandlung-von-zj-und-deren.pdf.

[15] Riedel A. (2015). Vertiefung von Ethik-Kompetenzen: Die Entwicklung einer Ethik-Leitlinie als methodisch-didaktische und strukturierende Rahmung. PADUA.

[16] Marckmann G., Schmidt H., Sofear N., Strech D. (2015). Putting public health ethics into practice: A systematic framework. Front. Public Health.

[17] Marckmann G. (2013). Wann ist eine ethische Analyse eine *gute* ethische Analyse? Ein Plädoyer für die Methodenreflexion in der Medizinethik. Ethik Med..

[18] Berlinger N., Jennings B., Wolf S.M. (2013). The Hastings Center Guidelines for Decisions on Life-Sustaining Treatment and Care Near the End of Life.

[19] Schweizerische Akademie der Medizinischen Wissenschaften (SAMW) (2012). Ethische Unterstützung in der Medizin. Schweiz. Ärztezeitung.

[20] Winkler E.C., Hiddemann W., Marckmann G. (2012). Evaluating a patient’s request for life-prolonging treatment: An ethical framework. J. Med. Ethics.

[21] Winkler E.C. (2008). Zur Ethik von ethischen Leitlinien: Sind sie die richtige Antwort auf moralisch schwierige Entscheidungssituationen im Krankenhaus und warum sollen Ärzte sie befolgen?. Z. Med. Ethik.

[22] Winkler E. (2005). The ethics of policy writing: How schould hospitals deal with moral disagreement about controversial medical practices?. J. Med. Ethics.

[23] Ledermann Flamm A., Hester D.M., Schonfeld T. (2012). Developping effective ethics policy. Guidance for Helthcare Ehics Committees.

[24] Frolic A.N., Drolet K. (2013). Ethics policy review: A case study in quality improvement. J. Med. Ethics.

[25] Riedel A., Dinges S., Fahr U., May A.T. (2013). Empfehlungen zur Evaluation von Ethikberatung in Einrichtungen des Gesundheitswesens. Ethik Med..

[26] International Council of Nurses (ICN) The ICN Code of Ethics for Nurses. http://www.dsr.dk/ser/documents/icncode_english.pdf.

[27] American Nurses Association (ANA) (2015). Code of Ethics for Nurses with Interpretive Statements.

[28] Richardson J., Heidenreich T., Álvarez-Nieto C., Fasseur F., Grose J., Huss N., Huynen M., López-Medina I.M., Schweizer A. Including Sustainability Issues in Nurse Education: A Comparative Study of First Year Student Nursus’ Attitudes in Four European Countries. http://dx.doi.org/10.1016/j.nedt.2015.11.005.

[29] Riedel A., Huber J.M., Linde A.C. (2013). Wiederkehrende ethische Dilemmata strukturiert reflektieren. Psych. Pflege.

